# Prenatal Bisphenol A Exposure and Early Childhood Behavior

**DOI:** 10.1289/ehp.0900979

**Published:** 2009-10-06

**Authors:** Joe M. Braun, Kimberly Yolton, Kim N. Dietrich, Richard Hornung, Xiaoyun Ye, Antonia M. Calafat, Bruce P. Lanphear

**Affiliations:** 1 Department of Epidemiology, University of North Carolina–Chapel Hill, Chapel Hill, North Carolina, USA; 2 Department of Pediatrics, Division of General and Community Pediatrics, Cincinnati Children’s Hospital Medical Center, Cincinnati, Ohio, USA; 3 Department of Environmental Health, Division of Epidemiology and Biostatistics, University of Cincinnati College of Medicine, Cincinnati, Ohio, USA; 4 Division of Laboratory Sciences, National Center for Environmental Health, Centers for Disease Control and Prevention, Atlanta, Georgia, USA; 5 Child and Family Research Institute, BC Children’s Hospital and the Faculty of Health Sciences, Simon Fraser University, Vancouver, British Columbia, Canada

**Keywords:** bisphenol A, children, endocrine, epidemiology, neurodevelopment

## Abstract

**Background:**

Prenatal exposure to bisphenol A (BPA) increases offspring aggression and diminishes differences in sexually dimorphic behaviors in rodents.

**Objective:**

We examined the association between prenatal BPA exposure and behavior in 2-year-old children.

**Methods:**

We used data from 249 mothers and their children in Cincinnati, Ohio (USA). Maternal urine was collected around 16 and 26 weeks of gestation and at birth. BPA concentrations were quantified using high-performance liquid chromatography–isotope-dilution tandem mass spectrometry. Child behavior was assessed at 2 years of age using the second edition of the Behavioral Assessment System for Children (BASC-2). The association between prenatal BPA concentrations and BASC-2 scores was analyzed using linear regression.

**Results:**

Median BPA concentrations were 1.8 (16 weeks), 1.7 (26 weeks), and 1.3 (birth) ng/mL. Mean (± SD) BASC-2 externalizing and internalizing scores were 47.6 ± 7.8 and 44.8 ± 7.0, respectively. After adjustment for confounders, log_10_-transformed mean prenatal BPA concentrations were associated with externalizing scores, but only among females [β = 6.0; 95% confidence interval (CI), 0.1–12.0]. Compared with 26-week and birth concentrations, BPA concentrations collected around 16 weeks were more strongly associated with externalizing scores among all children (β = 2.9; 95% CI, 0.2–5.7), and this association was stronger in females than in males. Among all children, measurements collected at ≤ 16 weeks showed a stronger association (β = 5.1; 95% CI, 1.5–8.6) with externalizing scores than did measurements taken at 17–21 weeks (β = 0.6; 95% CI, −2.9 to 4.1).

**Conclusions:**

These results suggest that prenatal BPA exposure may be associated with externalizing behaviors in 2-year-old children, especially among female children.

Bisphenol A (BPA) is a weak estrogenic monomer used to produce plastic polymers such as polycarbonate plastics and epoxy resins that can be found in toys, water supply pipes, medical tubing, and food container linings. More than 6 billion pounds of BPA were manufactured worldwide in 2003, making it one of the highest production-volume chemicals (Burridge 2003). Polymers made from BPA can be hydrolyzed in high temperature, acidic, and basic conditions, leading to BPA leaching into water and food containers and baby feeding bottles (Brede et al. 2003; Factor 1996; Nerin et al. 2003; Tan and Mustafa 2003).

The ubiquity of BPA-containing plastics makes human exposure nearly universal in developed countries. More than 90% of the U.S. population has detectable levels of urinary BPA (Calafat et al. 2008). BPA has also been detected in the urine and serum of pregnant women (Padmanabhan et al. 2008; Schonfelder et al. 2002; Wolff et al. 2008; Ye et al. 2008) and serum, plasma, and placenta of newborn infants (Lee et al. 2008; Schonfelder et al. 2002).

Given the role that estrogen and other aromatizable androgens play in neuronal connectivity, distribution of serotinergic fibers, synaptic function, and dendritic length, exposure to endocrine-disrupting compounds such as BPA may alter the course of normal neurodevelopment (Palanza et al. 1999). Prenatal BPA exposure in rodent studies is associated with increased aggression, morphine- and methamphetamine-induced hyperlocomotion, and memory impairment (Carr et al. 2003; Kawai et al. 2003; Miyagawa et al. 2007; Narita et al. 2006; Suzuki et al. 2003). Some rodent studies suggest that sex modifies the effect of prenatal BPA exposure (Adriani et al. 2003; Carr et al. 2003; Dessi-Fulgheri et al. 2002; Fujimoto et al. 2006; Kubo et al. 2001), whereas others do not (Farabollini et al. 2002; Nagao et al. 1999). The direction of the interaction between sex and BPA exposure is not clear because most of these studies have used different BPA doses, routes of exposure, and end points (Chapin et al. 2008).

To date, no studies have examined the association between prenatal BPA exposure and neurodevelopment in children (Chapin et al. 2008). Given the near universal exposure to BPA among the general U.S. population and the association between BPA exposure and neurodevelopment and behavior in animal studies, even small effects of BPA on childhood neurodevelopment could have profound public health impacts (Bellinger 2004b; Needleman 1990).

We used data from the Health Outcomes and Measures of the Environment Study to examine the association between prenatal BPA exposure and behavior in 2-year-old children. We also investigated whether sex modified the association between prenatal BPA exposure and childhood behavior.

## Methods

### Study sample

Data were collected from mothers and their children participating in the Health Outcomes and Measures of the Environment Study, an ongoing prospective birth cohort in the Cincinnati, Ohio (USA), metropolitan area designed to examine low-level environmental toxin exposure and the efficacy of injury and lead hazard controls in the home (Dietrich et al. 2005). Beginning in March 2003, women were identified from seven prenatal clinics associated with three hospitals. Eligibility criteria for the study included ≤ 19 weeks of gestation, age ≥ 18 years, living in a house built before 1978, negative HIV status, and not taking medications for seizure or thyroid disorders. We mailed letters to 5,184 women who were ≥ 18 years of age and living in a house built before 1978. Of the 1,263 eligible women, 468 provided informed consent and enrolled in the study.

### Maternal urinary BPA concentrations

Women provided three spot urine samples collected at approximately 16 and 26 weeks of gestation and at birth. Sixteen-week urine samples were collected when women were met by study staff at their prenatal care appointments; 26-week urine samples were collected when women received their glucose tolerance test for gestational diabetes screening. Women could have had their urine samples taken anytime during the screening test. Birth urine samples were typically collected before or within 24 hr of parturition.

Urine was collected in polyethylene containers and stored at −20°C until shipped to the Centers for Disease Control and Prevention (CDC) Environmental Health Laboratories, where they were stored at or below −20°C. Some urine samples were stored for 4–5 years before analysis. Recent work from the CDC laboratories suggests that urinary BPA species are stable for at least 6 months (Ye et al. 2007) and even as long as 30 months (Calafat et al. 2009) when the urine sample is stored at subfreezing temperatures.

The concentration of total (free plus conjugated) species of urinary BPA was quantified using modified analytical methods previously described (Ye et al. 2005a, 2005b). Urine samples were treated with β‐glucuronidase/sulfatase to hydrolyze conjugated BPA species. After hydrolysis, urine samples were acidified and then BPA was preconcentrated using online solid-phase extraction. BPA concentrations were quantified using high-performance liquid chromatography–isotope-dilution tandem mass spectrometry. The limit of detection (LOD) was 0.4 μg/L. Concentrations below the LOD were given a value of 
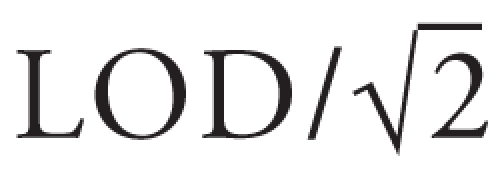
– for statistical analyses (Hornung and Reed 1990). Four quality control (QC) samples were analyzed in each analytic run. The low-concentration QC samples (~ 2.8 μg/L) had a coefficient of variation (CV) of 6.9–9.2%, whereas the high-concentration QC samples (~ 9.7 μg/L) had a CV of 3.4–7.6%.

### Childhood behavior

We assessed children’s behavior at 2 years of age using the second edition of the Behavioral Assessment System for Children (BASC-2) Parent Rating Scale for preschoolers (Reynolds and Kamphaus 2002).The BASC-2 is a 134-item parent-reported assessment of a child’s adaptive and problem behaviors in community and home settings. The preschool version is appropriate for children 2–5 years of age and has excellent reliability and validity for assessing adaptive and maladaptive behaviors (Ostrander et al. 1998; Reynolds and Kamphaus 2002). Composite scores are derived by the publisher’s computerized scoring program for externalizing, internalizing, and Behavior Symptom Index (BSI) scales, each with clinical subscales. Scores were normalized to a mean (± SD) of 50 ± 10 during the national standardization of the instrument. On the clinical behavioral subscales, scores in the range of 60–69 are considered “at risk,” and scores ≥ 70 are considered clinically significant. Composite scores for externalizing behaviors consist of hyperactivity and aggression scales, and internalizing composite scores consist of depression, anxiety, and somatization scales. The BSI is a composite score that reflects the overall level of problem behaviors and consists of scales for aggression, hyperactivity, depression, and attention. We used the general combined sex-normalized scores for our analyses. Creatinine-adjusted prenatal BPA concentrations were not qualitatively or statistically different between children with and without BASC-2 scores.

The BASC-2 uses the F-index as a measure of a parent’s tendency to be overly negative when describing their child. BASC-2 assessments with F-index scores > 3.0 are to be interpreted cautiously; however, in this study no BASC-2 assessments at 2 years of age had F-index ratings > 3.0.

### Confounders

We used directed acyclic graphs (DAGs) to examine the role of potential confounders in our exposure–disease relation (Greenland and Brumback 2002; Greenland et al. 1999). Simulation studies have shown that DAGs are a better method to assess the role of confounding variables than are change in estimate and significance testing approaches (Greenland 2008). Because little is known about factors associated with BPA exposures, we adjusted for many of the same confounders that are thought to confound other environmental toxin–neurodevelopment relations (Bellinger 2004a), including maternal and child demographic factors such as maternal age (< 25, 25–34, and ≥ 35 years), race (white and nonwhite), education (< 12, 12, and > 12 years), marital status (married and nonmarried), annual household income (< $40,000, $40,000 to < $80,000, ≥ $80,000), and child sex. Maternal depression during pregnancy was measured at 20 weeks of gestation with the second edition of the Beck Depression Inventory (BDI-II) (Beck et al. 1996). Women were classified as minimally or mildly depressed (BDI-II score < 20) and moderately or severely depressed (BDI-II score ≥ 20). Caregiving environment was measured by administering the Home Observation for Measurement of the Environment (HOME) Inventory during a 12-month home visit (Caldwell and Bradley 2003). HOME scores were categorized as < 35, 35–39, and ≥ 40 based on the distribution of the scores.

### Statistical analysis

Demographic characteristics of the 389 mothers and their singleton children were compared with the 249 dyads with complete urinary BPA and BASC-2 data. Descriptive statistics of BPA concentrations at each time point were calculated along with the proportion of measurements below the LOD. We also calculated the Pearson correlation coefficient between the nonadjusted and creatinine-adjusted BPA concentrations at each time point.

We examined the shape of the association between maternal BPA concentration and children’s behavior using graphical and locally weighted scatterplot smoothing (LOESS) analysis. LOESS uses locally weighted polynomials to fit subsets of data using ordinary least squares regression, giving more weight to points closer to the point being estimated, and less weight to points farther away (Cleveland 1979).

We started our analyses by examining the association between the mean prenatal BPA concentration (i.e., the mean of 16- and 26-week and birth BPA concentrations) and externalizing, internalizing, and BSI scores using multivariable linear regression. We used continuous log_10_-transformed mean BPA concentrations and quartiles of the mean BPA concentration in separate models. Coefficients from the continuous models represent the mean change in BASC-2 scores for a 10-fold increase in BPA concentrations. Adjusted mean scores for each quartile were calculated, as well as the difference between the first quartile mean score and the other three quartiles’ mean score.

Next, we analyzed the association between each individual BPA measurement and externalizing, internalizing, and BSI scores to examine whether the association was dependent on the timing of exposure. We constructed models using each log_10_-transformed BPA concentration and quartiles of BPA concentration as the independent variable.

Finally, we examined the association between early prenatal BPA exposure and BASC-2 scores, by looking for differences in the association between BPA concentrations and BASC-2 scores based on the timing of the 16-week urine measurement. Each individual woman had only one urine sample taken at approximately 16 weeks of gestation. We estimated the gestational week of the 16-week measurement using women’s reported last menstrual period (LMP). We dichotomized the timing of the 16-week urine measurement at the median value of 16 weeks. We included a product interaction term between the log_10_-transformed BPA concentration and the dichotomous measurement time variable. This allowed us to estimate the association between BPA concentrations and BASC-2 scores separately for women with measurements ≤ 16 weeks of gestation and > 16 weeks of gestation.

Based on previous animal literature suggesting that BPA may be associated with disruptions in sexually dimorphic behaviors, we estimated sex-specific associations between BPA concentrations and BASC-2 scores by including a product interaction term between the BPA concentration and child sex variables. Male- and female-specific slope coefficients were computed from regression models using continuous BPA concentration, whereas sex-specific means and differences were calculated from models using creatinine-adjusted BPA concentration quartiles. We assessed for the presence of sex modification by examining the *p*-value for the interaction term between BPA concentration and sex. We considered *p*-values < 0.10 to be indicative of effect measure modification (Rothman and Greenland 1998).

We used urinary creatinine concentrations to control for urine dilution in all of our analyses. In models using continuous mean prenatal BPA concentrations, each individual BPA concentration was divided by the respective urinary creatinine concentration before taking the mean of the measurements. In models using individual BPA concentrations, we included the log_10_-transformed urinary creatinine value from the respective measurement in our models. In analyses involving percentiles, we created percentiles from the distribution of creatinine-adjusted BPA concentrations. We excluded 10, 20, and 15 women with urinary creatinine values < 20 mg/dL at the 16-week, 26-week, and birth measurement, respectively, to reduce the influence of values from very dilute urine (Wolff et al. 2008).

### Secondary analyses

Several sets of secondary analyses were conducted. First, we sought to determine if mean and time-specific prenatal (16 and 26 weeks of gestation and birth) serum cotinine or blood lead levels confounded the association between BPA concentrations and BASC-2 scores. Concurrent log_10_-transformed serum cotinine or blood lead levels were entered into respective log_10_-transformed continuous BPA models (i.e., 16-week cotinine included in 16-week BPA model). We also ran models that included both serum cotinine and blood lead levels. Second, we conducted an additional analysis that included Full Scale maternal IQ (Wechsler Abbreviated Scales of Intelligence) as a continuous variable in our model (Wechsler 1999). We also conducted additional analyses that included women with urinary creatinine concentrations < 20 mg/dL to see whether this restriction changed our estimates. All analyses were conducted with SAS version 9.2 (SAS Institute Inc., Cary, NC, USA).

### Ethical considerations

The institutional review boards (IRBs) of the University of North Carolina–Chapel Hill, Cincinnati Children’s Hospital Medical Center, and CDC approved this study. The University of Cincinnati College of Medicine IRB was involved in the oversight of this study throughout all stages of planning and implementation. All mothers provided written informed consent for themselves and their children before enrollment in the study.

## Results

Of the 468 women who enrolled in our study, 67 dropped out before delivery. We excluded nine sets of twins, and three stillborn children. Among the 389 women with singleton live births, all three urine samples were collected from 332 (85.3%) women. BASC-2 scores were available for 249 (64.0%) of the children whose mothers had all three urinary BPA measurements. Women from dyads with complete BPA and BASC-2 data were more likely to be white, better educated, wealthier, married, and 25–34 years of age than women in the original sample (Table 1). Among the 249 children born to women with all three prenatal BPA concentrations, the mean (± SD) externalizing, internalizing, and BSI scores were 47.6 ± 7.8 (range, 30–84), 44.8 ± 7.0 (range, 31–68), and 50.5 ± 6.8 (range, 34–74), respectively.

Almost all (*n* = 247, 99.2%) women had at least one urine sample with a detectable urinary BPA concentration (Table 2; 16 weeks, 89.2%; 26 weeks, 89.6%; birth, 87.1%). One woman’s 26-week urine BPA concentration was 3 orders of magnitude higher than the median 26-week concentration. Excluding this woman’s results did not substantially alter our effect estimates. Creatinine-adjusted BPA concentration distributions were very similar across pregnancy and at birth. Log_10_-transformed BPA concentrations were modestly correlated at 16 and 26 weeks (Pearson *r* = 0.30), 16 weeks and birth (Pearson *r* = 0.26), and 26 weeks and birth (Pearson *r* = 0.28). We found little correlation between log_10_-transformed creatinine-adjusted BPA concentrations at 16 and 26 weeks (Pearson *r* = 0.11), 16 weeks and birth (Pearson *r* = 0.11), and 26 weeks and birth (Pearson *r* = 0.08)

LOESS analysis revealed linear associations between mean and individual urinary BPA concentrations and BASC-2 scores. We found no association between mean prenatal BPA concentrations and externalizing, internalizing, or BSI scores for the entire sample. In sex-stratified analyses, we observed a positive association among females between log_10_-transformed mean prenatal BPA concentrations and externalizing [β = 6.0; 95% confidence interval (CI), 0.1–12.0] and BSI scores (β = 5.5; 95% CI, 0.3–10.7), but not among males (*p*-value for interaction < 0.05; Table 3).

Covariate and creatinine-adjusted log_10_-transformed 16-week BPA concentrations were positively associated with externalizing scores (Table 4; β = 2.9; 95% CI, 0.2–5.7). The association between continuous 16-week BPA concentrations and externalizing scores was stronger among females (β = 4.8; 95% CI, 1.3–8.3) than for males (β = 1.1; 95% CI, −2.4 to 4.6). Sex also modified the association between 16-week BPA maternal concentrations and children’s BSI scores. Maternal BPA concentrations at 26 weeks were not associated with children’s externalizing behaviors. We found a modest inverse association between maternal 26-week BPA concentration and children’s internalizing and BSI scores (β = −2.2; 95% CI, −4.6 to 0.7).

Children born from women in the top three quartiles of birth BPA concentrations had mean externalizing scores approximately 2 points higher than those of children from women in the lowest quartile. Males born from women in the second and third quartiles of BPA concentration had higher mean internalizing and BSI scores compared with males born to women in the first quartile.

**Figure 1:**
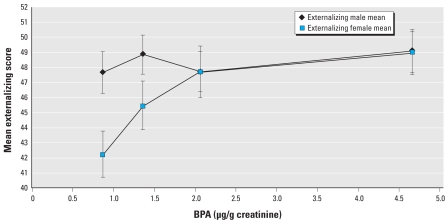
Mean ± SE adjusted externalizing BASC-2 score by median 16-week BPA quartile, stratified by child sex. Data are adjusted for maternal age (< 25, 25–34, and ≥ 35 years), race (white and nonwhite), maternal education (< 12 years, 12 years, > 12 years), household income (< $40,000, $40,000 to < $80,000, ≥ $80,000), HOME score (< 35, 35 to < 40, and ≥ 40), marital status (married vs. unmarried), and maternal depression during pregnancy (minimal/mild vs. moderate/severe).

Mean externalizing scores increased across quartiles of maternal creatinine-adjusted 16-week BPA quartile among female children, whereas externalizing scores did not differ among males (Figure 1). Externalizing scores among females in the third and fourth quartiles were comparable to mean externalizing scores for males.

The timing of the 16-week urine measurement modified the association between BPA concentrations and externalizing scores. A total of 242 women had LMP-based estimates of the 16-week measurement. Among these women, 134 (55.4%) had their urine measurements taken at ≤ 16 weeks of gestation (minimum, 11.2 weeks) and 108 (44.6%) had their measurements taken at > 16 weeks (maximum, 21.4 weeks). Confounder-adjusted log_10_-transformed BPA concentrations taken at ≤ 16 weeks of gestation were positively associated (β = 5.1; 95% CI, 1.5–8.6) with externalizing scores, whereas those taken at > 16 weeks of gestation were not (β = 0.6; 95% CI, −2.9 to 4.1). The positive association between BPA concentrations and externalizing behaviors was stronger in females (β = 7.0; 95% CI, 3.0–11.0; *n* = 70) than in males (β = 3.0; 95% CI, –1.0 to 7.0; *n* = 64) among mothers whose urine samples were taken at ≤ 16 weeks of gestation (*p*-value for BPA × sex interaction term < 0.10). We found no qualitative or statistical differences in the mean log_10_-transformed creatinine-adjusted BPA concentrations taken at ≤ 16 weeks and > 16 weeks. The proportion of males and females was not qualitatively or statistically different by the timing of the 16-week measurement.

### Secondary analyses

In secondary analyses, the inclusion of maternal blood lead levels, maternal serum cotinine levels, or both did not substantially alter our results. The inclusion of maternal IQ did not substantially alter any of our findings and was not associated with BASC-2 scores. Including women with urinary creatinine values < 20 mg/dL did not substantially change the estimates in any of our analyses. Our results were unchanged when we conducted analyses that included women with one or two missing BPA measurements.

## Discussion

This is the first study to examine the association between prenatal BPA exposure and childhood behavior. We did not observe an association between mean urinary BPA concentrations and behavior scores among all children. However, we report an association between mean prenatal BPA concentrations and externalizing scores in females. Further examination revealed that the association between prenatal BPA concentrations and externalizing behaviors in 2-year-old girls was driven by maternal urine BPA concentrations measured at 16 weeks of gestation. The association between early BPA concentrations and externalizing scores was strongest among women with urine measurements taken at ≤ 16 weeks of gestation. The interaction between sex and 16-week BPA concentrations persisted in samples taken at ≤ 16 weeks of gestation.

Consistent with our findings, some animal studies have shown that prenatal BPA exposure is associated with increased aggression (Kawai et al. 2003), changes in the dopaminergic system in the limbic forebrain (Narita et al. 2006; Suzuki et al. 2003), and other neurobehavioral changes (Carr et al. 2003; Kawai et al. 2003; Miyagawa et al. 2007; Narita et al. 2006; Suzuki et al. 2003). To our knowledge, only one rodent study has examined for time-sensitive periods of BPA exposure. Narita et al. (2007) reported that BPA exposure during organogenesis and breast-feeding caused morphine-induced hyperlocomotion, preference for drug-paired place, and up-regulation of dopamine receptor function, whereas exposure at other gestational time periods did not.

We observed an inverted U-shaped association between maternal urinary BPA concentrations and internalizing behaviors among male children in our sample. Others have written extensively about the possibility of a nonlinear dose–response curve between environmental estrogens and various end points (Welshons et al. 2003).

Our results suggest that child sex may modify the association between prenatal BPA exposure and externalizing behaviors. Interactions between child sex and prenatal exposure to neurodevelopmental toxicants have been observed in some epidemiologic studies (Bellinger et al. 1990; Engel et al. 2009; Pocock et al. 1987; Ris et al. 2004; Vreugenhil et al. 2002); however, sex-specific effect measure modification is usually not reported in epidemiologic studies. Results from rodent studies have reported that sex modifies the effect of prenatal BPA exposure (Adriani et al. 2003; Dessi-Fulgheri et al. 2002; Fujimoto et al. 2006; Kubo et al. 2001) and that prenatal BPA exposure may abolish or reverse differences in sexually dimorphic behaviors (Palanza et al. 2008). However, it is difficult to label effects as feminizing or masculinizing without knowing whether these end points are sexually dimorphic (Li et al. 2008).

Two issues must be considered when comparing the results of our study to those of rodent studies. First, the route of exposure in animal studies has included oral, subcutaneous, and direct injection at target organs (Li et al. 2008), whereas humans are exposed predominantly through the oral route (Chapin et al. 2008). Second, animal studies have used doses spanning several orders of magnitude, many of which are not relevant to human exposure (Li et al. 2008).

Prenatal urinary BPA concentrations among women in this study were similar to those from women of childbearing age from a nationally representative U.S. sample (Calafat et al. 2008), from a previous study examining the association between BPA exposure and infant birth outcomes among minority pregnant women in New York City (Wolff et al. 2008), and from pregnant women in Rotterdam, the Netherlands (Ye et al. 2008).

This study has several limitations. First, the children in our sample were 2 years of age at the time of behavioral assessment. Patterns of behavior are variable during early childhood. However, longitudinal analyses of children reveal that inattentive/hyperactive behaviors tend to be stable over childhood, whereas aggressive behaviors decrease (Jester et al. 2005; Nagin and Tremblay 2001; Tremblay et al. 2004). Second, unmeasured confounding may be responsible for some or all of our observed associations. Mink et al. (2004) have shown that unmeasured confounding can be responsible for much of the observed association between environmental neurotoxicants and neurobehavioral function. Although we included many of the same confounders used in studies of other neurotoxicants, it is possible that we did not adequately assess parental psychopathology (e.g., parental attention deficit/hyperactivity disorder). Third, there is evidence that prenatal BPA exposure disrupts normal mother–child interactions in rodent models (Palanza et al. 2008). Female mice exposed to BPA spend less time in their nest and nursing their offspring and more time grooming and resting alone. Therefore, associations between childhood behavior and prenatal BPA exposure may be mediated through maternal behavior toward the child. Finally, maternal BPA measurements taken at 26 weeks and birth may be influenced by the glucose tolerance test and birthing process, respectively. However, this is unlikely because creatinine-adjusted BPA concentration distributions were almost identical at each time point. We would have expected BPA concentrations to be higher at birth because women would be more likely to be exposed to BPA containing plastics, such as intravenous tubing, in the hospital setting (Calafat et al. 2009). The lack of a difference could be attributable to increased plasma volume from intravenous fluids, hospital diet or fasting near delivery, metabolic changes from the birthing process, hospital policies that limit BPA-containing plastics, or a combination of all of these factors.

Exposure misspecification may be responsible for our observed results. This could bias our results toward or away from the null, depending on the nature of the misspecification and characterization of the exposure (continuous vs. categorical). BPA has a short half-life in biological tissues (Volkel et al. 2002), although a recent analysis of the 2003–2004 National Health and Nutrition Examination Survey suggests that BPA may have a longer than expected half-life (Stahlhut et al. 2009). Urinary BPA concentrations have a moderate degree of intraindividual variability, making it difficult to accurately characterize exposure from a single measurement. The lack of prior literature on the appropriate specification of BPA exposure during pregnancy makes it difficult to determine if a single measurement or summary measurement of BPA concentration more accurately quantifies exposure. Because women did not have repeated urine measurements around 16 weeks of gestation, we assumed that timing of the 16-week urine measurement was random. The results of these analyses should be interpreted cautiously because this assumption may not be true. As recommended previously by Wolff et al. (2008), additional research into characterizing BPA exposure is necessary. An additional source of exposure misspecification may have arisen from BPA degradation in samples that were stored for > 3 years. However, prior work indicates that storing urine samples at subfreezing temperatures for as long as 30 months does not result in substantial loss of BPA (Calafat et al. 2009).

Another limitation is that we were not able to examine whether postnatal BPA exposure was associated with childhood behavior. We are planning to examine this association in future studies of this cohort.

Selection bias is another potential limitation of our study because women were required to be living in pre-1978 housing. It is possible that children living in older housing stock come from lower socioeconomic status, making them more likely to have behavior problems. However, this is unlikely because families in our sample have higher levels of education and income than the general population.

Finally, there were two statistical limitations to this study. First, it could be argued that our observed associations are spurious because of the large number of associations we examined. However, multiple comparison arguments assume that randomness underlies the variability of all the observed associations (Rothman 1990; Savitz and Olshan 1995). It is unlikely that our results are attributable to random variation because we observed a consistent association between prenatal urinary BPA concentrations and externalizing behaviors in females that became stronger in measurements taken earlier in pregnancy. Second, our power to detect associations between maternal BPA exposure and childhood behavior was diminished in our analyses examining sex- and time-specific associations, resulting in imprecise estimates.

This study has several strengths. First, we had three urinary BPA measurements in the latter two-thirds of pregnancy to estimate gestational exposure. This allowed us to examine whether some periods of gestation are sensitive to BPA exposure. Second, we used a valid and reliable measure of adaptive and problem behaviors in children (Kamphaus et al. 1999). Finally, we are continuing to follow these children through 5 years of age, which will allow us to examine whether our observed associations persist throughout early childhood.

Mean prenatal BPA concentrations were not associated with childhood behavior. However, we did observe a positive association between these concentrations and externalizing behaviors among females. Early second-trimester maternal urinary BPA concentrations were positively associated with externalizing behaviors and global behavior scores among all children, but especially among 2-year-old female children. Our results suggest that BPA concentrations in samples taken earlier in pregnancy may be more strongly associated with externalizing scores than those in later samples. The reported associations and interactions between child sex and timing of BPA exposure should be viewed cautiously because these results could be biased by exposure misspecification or residual confounding. Future research should aim to develop methods to accurately characterize BPA exposure, especially during the first trimester of pregnancy; to validate the associations observed in this cohort; and to examine the association between pre- and postnatal BPA exposure and behavior in later childhood.

## Figures and Tables

**Table 1:** Demographic, maternal, and household characteristics of all mother–child dyads and those with all urine measurements and 2-year BASC-2 data in the HOME study [no. (%)].

Variable	All women and children (*n* = 389)	Women and children with all urine and behavior measurements (*n* = 249)
Child sex		
Female	208 (53.5)	131 (52.6)
Male	181 (46.5)	118 (47.4)
Missing	0	0

Maternal race		
White	237 (61.7)	180 (72.3)
Nonwhite	147 (38.3)	69 (27.7)
Missing	5	0

Maternal education (years)		
< 12	41 (10.7)	16 (6.4)
12	54 (13.8)	22 (8.8)
> 12	289 (74.5)	211 (84.7)
Missing	5	0

Annual household income category		
< $40,000	153 (40.7)	72 (29.6)
$40,000 to < $80,000	120 (31.9)	94 (38.7)
≥ $80,000	103 (27.4)	77 (31.7)
Missing	13	6

Maternal marital status		
Married	249 (64.8)	188 (75.5)
Single	135 (35.2)	61 (24.5)
Missing	5	0

Maternal age category (years)		
< 25	96 (24.7)	43 (17.3)
25–34	231 (59.4)	168 (67.5)
≥ 35	62 (15.9)	38 (15.3)
Missing	0	0

HOME score category (12 months)		
< 35	51 (15.3)	29 (11.8)
35–39	64 (19.2)	39 (15.8)
≥ 40	219 (65.6)	178 (72.4)
Missing	55	3

Maternal BDI-II at 20 weeks		
Minimal or mild depression (0–19)	345 (92.0)	230 (93.1)
Moderate or severe depression (≥ 20)	30 (8.0)	17 (6.9)
Missing	14	2

**Table 2:** Descriptive statistics of 16-week, 26-week, and birth urinary BPA concentrations among mothers enrolled in the HOME study.

			Percentile					
	*n* ≥ LOD (%)	Minimum^a^	5th	25th	50th	75th	95th	Maximum
No creatinine adjustment (μg/L)								
All children (*n* = 249)								
16 weeks	222 (89.2)	< LOD	< LOD	0.8	1.8	3.2	9.7	37.3
26 weeks	223 (89.6)	< LOD	< LOD	0.8	1.7	3.0	9.8	1,250
Birth	217 (87.1)	< LOD	< LOD	0.7	1.3	2.5	6.6	48.7
Mean	NA	< LOD	0.5	1.1	2.0	3.3	8.2	421
Males (*n* = 118)								
16 weeks	104 (88.1)	< LOD	< LOD	0.8	1.8	3.0	9.6	19.5
26 weeks	109 (92.4)	< LOD	< LOD	0.8	1.7	3.1	12.5	1,250
Birth	103 (87.3)	< LOD	< LOD	0.7	1.4	2.5	6.8	48.7
Mean	NA	< LOD	0.5	1.1	1.9	3.2	8.5	421
Females (*n* = 131)								
16 weeks	118 (90.1)	< LOD	< LOD	0.9	1.9	3.4	10.3	37.3
26 weeks	114 (87.0)	< LOD	< LOD	0.8	1.5	3.0	9.0	41.3
Birth	114 (87.0)	< LOD	< LOD	0.7	1.2	2.5	6.5	12.0
Mean	NA	< LOD	0.5	1.1	2.0	3.3	7.6	17.7

Creatinine adjusted (μg/g)								
All children (*n* = 249)								
16 weeks	222 (89.2)	< LOD	< LOD	1.1	1.6	3.0	22.2	34.8
26 weeks	223 (89.6)	< LOD	< LOD	1.3	2.0	3.1	8.0	583
Birth	217 (87.1)	< LOD	< LOD	1.2	1.9	2.9	9.2	27.3
Mean	NA	< LOD	1.0	1.6	2.2	3.4	8.0	196
Males (*n* = 118)								
16 weeks	104 (88.1)	< LOD	< LOD	1.0	1.5	3.0	9.3	19.4
26 weeks	109 (92.4)	< LOD	< LOD	1.3	2.1	3.1	10.0	583.0
Birth	103 (87.3)	< LOD	< LOD	1.2	1.7	2.6	9.6	27.3
Mean	NA	< LOD	0.9	1.6	2.1	3.3	7.8	196
Females (*n* = 131)								
16 weeks	118 (90.1)	< LOD	< LOD	1.1	1.8	3.0	10.7	34.8
26 weeks	114 (87.0)	< LOD	< LOD	1.3	2.0	3.1	6.2	32.8
Birth	114 (87.0)	< LOD	< LOD	1.2	2.0	3.1	9.2	15.8
Mean	NA	< LOD	1.1	1.6	2.3	3.4	8.0	13.9

NA, not applicable.

a LOD = 0.4 μg/L; 5th percentile values are 0.28 (
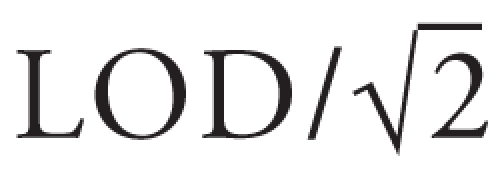
).

**Table 3:** Adjusted associations between mean prenatal creatinine-adjusted BPA concentration and externalizing, internalizing, and BSI BASC-2 scores among 2-year-old children in the HOME study.

	All children		Males		Females	
Score, BPA exposure	Adjusted score (mean ± SD)	Adjusted difference^a^ (95% CI)	Adjusted score (mean ± SD)	Adjusted difference^b^ (95% CI)	Adjusted score (mean ± SD)	Adjusted difference^b^ (95% CI)
Externalizing score						
First quartile (0–1.52 μg/g)	46.5 ± 1.1	Reference	48.4 ± 1.5	Reference	44.5 ± 1.5	Reference
Second quartile (1.53–2.10 μg/g)	46.6 ± 1.1	0.1 (−3.0 to 3.2)	49.4 ± 1.4	1.1 (−3.1 to 5.2)	43.2 ± 1.7	−1.4 (−5.9 to 3.1)
Third quartile (2.11–3.31 μg/g)	48.7 ± 1.1	2.2 (−0.9 to 5.3)	49.7 ± 1.7	1.3 (−3.2 to 5.9)	47.7 ± 1.4	3.1 (−1.0 to 7.3)
Fourth quartile (> 3.31 μg/g)	48.0 ± 1.1	1.5 (−1.6 to 4.6)	47.0 ± 1.5	−1.4 (−5.7 to 2.9)	48.9 ± 1.5	4.4 (0.1 to 8.6)*
Log_10_-transformed coefficient		1.0 (−2.7 to 4.7)		−1.9 (−6.4 to 2.7)		6.0 (0.1 to 12.0)**

Internalizing score						
First quartile (0–1.52 μg/g)	44.1 ± 1.0	Reference	43.3 ± 1.4	Reference	44.9 ± 1.4	Reference
Second quartile (1.53–2.10 μg/g)	44.2 ± 1.0	0.1 (−2.7 to 2.8)	45.1 ± 1.3	1.8 (−1.9 to 5.6)	42.9 ± 1.5	−2.0 (−6.1 to 2.1)
Third quartile (2.11–3.31 μg/g)	44.5 ± 1.0	0.4 (−2.4 to 3.1)	45.4 ± 1.5	2.2 (−2.0 to 6.3)	43.9 ± 1.2	−0.9 (−4.7 to 2.8)
Fourth quartile (> 3.31 μg/g)	44.4 ± 1.0	0.3 (−2.5 to 3.1)	42.3 ± 1.4	−0.9 (−4.8 to 3.0)	46.5 ± 1.4	1.6 (−2.3 to 5.5)
Log_10_-transformed coefficient		−0.4 (−3.7 to 2.9)		−2.1 (−6.2 to 2.0)		2.8 (−2.7 to 8.2)

BSI score						
First quartile (0–1.52 μg/g)	50.5 ± 1.0	Reference	52.5 ± 1.3	Reference	48.3 ± 1.4	Reference
Second quartile (1.53–2.10 μg/g)	49.5 ± 1.0	−1.0 (−3.7 to 1.7)	51.8 ± 1.2	−0.7 (−4.4 to 3.0)	46.8 ± 1.5	−1.5 (−5.5 to 2.4)
Third quartile (2.11–3.31 μg/g)	50.9 ± 1.0	0.4 (−2.3 to 3.1)	51.9 ± 1.5	−0.6 (−4.6 to 3.4)	49.8 ± 1.2	1.5 (−2.1 to 5.1)
Fourth quartile (> 3.31 μg/g)	50.5 ± 1.0	0.0 (−2.7 to 2.7)	49.3 ± 1.4	−3.2 (−7.0 to 0.6)	51.5 ± 1.3	3.2 (−0.6 to 6.9)**
Log_10_-transformed coefficient		0.0 (−3.3 to 3.2)		−3.1 (−7.1 to 0.8)		5.5 (0.3 to 10.7)**

a Adjusted for maternal age (< 25, 25–34, and ≥ 35 years), child sex, race (white and nonwhite), maternal education (< 12 years, 12 years, > 12 years), household income (< $40,000, $40,000 to < $80,000, ≥ $80,000), HOME score (< 35, 35 to < 40, and ≥ 40), marital status (married vs. unmarried), and maternal depression during pregnancy (minimal/mild vs. moderate/ severe).

b Adjusted for maternal age (< 25, 25–34, and ≥ 35 years), race (white and nonwhite), maternal education (< 12 years, 12 years, > 12 years), household income (< $40,000, $40,000 to < $80,000, ≥$80,000), HOME score (< 35, 35 to < 40, and ≥ 40), marital status (married vs. unmarried), and maternal depression during pregnancy (minimal/mild vs. moderate/severe).

* *p-*Value < 0.10 for the interaction term between BPA concentration and child sex.

** *p*-Value < 0.05 for the interaction term between BPA concentration and child sex.

**Table 4:** Adjusted associations between individual creatinine-adjusted BPA concentrations and BASC-2 externalizing, internalizing, and BSI scores among 2-year-old children participating in the HOME study.

	All children^a^		Males^b^		Females^b^	
Score	Adjusted estimate (SE)	Adjusted difference (95% CI)	Adjusted estimate (SE)	Adjusted difference (95% CI)	Adjusted estimate (SE)	Adjusted difference (95% CI)
16-week BPA concentrations	*n* = 229		*n* = 113		*n* = 116	

Externalizing scores						
First quartile (0–1.09 μg/g)	45.2 (1.1)	Reference	47.7 (1.4)	Reference	42.2 (1.5)	Reference
Second quartile (1.10–1.62 μg/g)	47.2 (1.1)	2.1 (−0.7 to 4.8)	48.9 (1.3)	1.2 (−2.4 to 4.8)	45.5 (1.6)	3.2 (−0.9 to 7.4)
Third quartile (1.63–2.88 μg/g)	48.1 (1.1)	2.9 (0.2 to 5.7)	47.7 (1.7)	0.0 (−4.1 to 4.2)	47.8 (1.3)	5.5 (1.8 to 9.3)*
Fourth quartile (> 2.88 μg/g)	49.0 (1.0)	3.9 (1.1 to 6.6)	49.1 (1.4)	1.4 (−2.3 to 5.2)	49.0 (1.4)	6.7 (2.7 to 10.7)*
Log_10_-transformed coefficient^c^		2.9 (0.2 to 5.7)		1.1 (−2.4 to 4.6)		4.8 (1.3 to 8.3)*
Internalizing scores						
First quartile (0–1.09 μg/g)	44.6 (1.0)	Reference	44.6 (1.3)	Reference	44.4 (1.4)	Reference
Second quartile (1.10–1.62 μg/g)	43.3 (1.1)	−1.2 (−3.8 to 1.3)	42.7 (1.2)	−1.9 (−5.3 to 1.4)	44.1 (1.5)	−0.3 (−4.2 to 3.5)
Third quartile (1.63–2.88 μg/g)	44.0 (1.0)	−0.5 (−3.1 to 2.0)	44.2 (1.6)	−0.4 (−4.3 to 3.4)	44.1 (1.2)	−0.3 (−3.8 to 3.2)
Fourth quartile (> 2.88 μg/g)	45.8 (1.0)	1.2 (−1.3 to 3.8)	45.0 (1.3)	0.3 (−3.2 to 3.8)	46.6 (1.3)	2.3 (−1.5 to 6.0)
Log_10_-transformed coefficient^c^		1.1 (−1.4 to 3.6)		0.0 (−3.2 to 3.3)		2.3 (−1.0 to 5.5)
BSI scores						
First quartile (0–1.09 μg/g)	49.0 (1.0)	Reference	51.6 (1.2)	Reference	45.8 (1.4)	Reference
Second quartile (1.10–1.62 μg/g)	49.7 (1.0)	0.8 (−1.7 to 3.2)	51.1 (1.2)	−0.5 (−3.7 to 2.7)	48.3 (1.4)	2.5 (−1.2 to 6.1)
Third quartile (1.63–2.88 μg/g)	50.9 (1.0)	1.9 (−0.5 to 4.4)	50.9 (1.5)	−0.7 (−4.4 to 3.0)	50.4 (1.2)	4.6 (1.3 to 7.9)**
Fourth quartile (> 2.88 μg/g)	51.0 (0.9)	2.1 (−0.4 to 4.5)	50.3 (1.3)	−1.3 (−4.7 to 2.0)	51.8 (1.2)	6.0 (2.4 to 9.5)**
Log_10_-transformed coefficient^c^		1.4 (−1.0 to 3.9)		−0.6 (−3.7 to 2.5)		3.5 (0.4 to 6.6)**

26-week BPA concentrations	*n* = 218		*n* = 106		*n* = 112	

Externalizing scores						
First quartile (0–1.24 μg/g)	47.5 (1.2)	Reference	48.8 (1.6)	Reference	46.3 (1.6)	Reference
Second quartile (1.25–1.97 μg/g)	48.1 (1.1)	0.5 (−2.4 to 3.5)	49.4 (1.6)	0.6 (−3.6 to 4.8)	46.8 (1.6)	0.4 (−3.7 to 4.6)
Third quartile (1.98–3.07 μg/g)	45.9 (1.2)	−1.6 (−4.6 to 1.3)	47.3 (1.5)	−1.6 (−5.8 to 2.6)	44.7 (1.6)	−1.7 (−5.8 to 2.5)
Fourth quartile (> 3.07 μg/g)	47.3 (1.1)	−0.2 (−3.2 to 2.8)	48.1 (1.6)	−0.7 (−5.0 to 3.6)	46.6 (1.5)	0.2 (−3.9 to 4.3)
Log_10_-transformed coefficient^c^		−1.0 (−3.9 to 1.9)		−1.4 (−4.8 to 2.0)		−0.4 (−4.5 to 3.8)
Internalizing scores						
First quartile (0–1.24 μg/gm)	45.3 (1.1)	Reference	44.3 (1.4)	Reference	46.2 (1.4)	Reference
Second quartile (1.25–1.97 μg/g)	43.6 (1.0)	−1.7 (−4.4 to 1.0)	42.7 (1.4)	−1.6 (−5.5 to 2.2)	44.3 (1.4)	−1.8 (−5.6 to 1.9)
Third quartile (1.98–3.07 μg/g)	44.5 (1.0)	−0.8 (−3.5 to 1.9)	45.1 (1.4)	0.8 (−2.9 to 4.6)	43.8 (1.4)	−2.4 (−6.2 to 1.3)
Fourth quartile (> 3.07 μg/g)	44.0 (1.0)	−1.3 (−4.0 to 1.4)	43.7 (1.4)	−0.6 (−4.5 to 3.2)	44.2 (1.3)	−2.0 (−5.7 to 1.7)
Log_10_-transformed coefficient^c^		−2.0 (−4.6 to 0.7)		−1.7 (−4.7 to 1.4)		−2.5 (−6.2 to 1.2)
BSI scores						
First quartile (0–1.24 μg/g)	51.3 (1.0)	Reference	51.7 (1.4)	Reference	51.0 (1.4)	Reference
Second quartile (1.25–1.97 μg/g)	50.6 (1.0)	−0.7 (−3.3 to 1.9)	51.5 (1.4)	−0.1 (−3.8 to 3.6)	49.6 (1.4)	−1.3 (−5.0 to 2.3)
Third quartile (1.98–3.07 μg/g)	48.6 (1.0)	−2.6 (−5.2 to 0.0)	50.4 (1.3)	−1.3 (−4.9 to 2.4)	47.0 (1.4)	−4.0 (−7.6 to −0.3)
Fourth quartile (> 3.07 μg/g)	49.7 (1.0)	−1.6 (−4.2 to 1.0)	51.1 (1.4)	−0.5 (−4.3 to 3.2)	48.5 (1.3)	−2.5 (−6.1 to 1.1)
Log_10_-transformed coefficient^c^		−2.2 (−4.7 to 0.4)		−1.9 (−4.9 to 1.1)		−2.6 (−6.3 to 1.0)

Birth BPA concentrations	*n* = 224		*n* = 112		*n* = 112	

Externalizing scores						
First quartile (0–1.16 μg/g)	46.1 (1.1)	Reference	47.3 (1.5)	Reference	45.0 (1.5)	Reference
Second quartile (1.17–1.80 μg/g)	48.2 (1.2)	2.1 (−0.8 to 5.0)	49.9 (1.5)	2.5 (−1.4 to 6.5)	46.7 (1.7)	1.6 (−2.6 to 5.8)
Third quartile (1.81–2.96 μg/g)	48.2 (1.1)	2.1 (−0.7 to 4.9)	49.7 (1.4)	2.4 (−1.6 to 6.3)	46.8 (1.6)	1.8 (−2.3 to 5.9)
Fourth quartile (> 2.96 μg/g)	48.1 (1.1)	2.0 (−0.8 to 4.9)	48.3 (1.6)	0.9 (−3.2 to 5.1)	47.9 (1.4)	2.8 (−1.1 to 6.8)
Log_10_-transformed coefficient^c^		1.8 (−1.2 to 4.8)		1.6 (−2.3 to 5.4)		2.1 (−2.0 to 6.2)
Internalizing scores						
First quartile (0–1.16 μg/g)	43.2 (1.0)	Reference	41.2 (1.4)	Reference	45.1 (1.4)	Reference
Second quartile (1.17–1.80 μg/g)	46.2 (1.1)	2.9 (0.2 to 5.6)	46.9 (1.3)	5.6 (2.0 to 9.2)	45.1 (1.5)	0.0 (−3.8 to 3.8)
Third quartile (1.81–2.96 μg/g)	45.5 (1.0)	2.3 (−0.3 to 4.9)	46.7 (1.3)	5.4 (1.8 to 9.1)	44.0 (1.4)	−1.1 (−4.8 to 2.7)
Fourth quartile (> 2.96 μg/g)	44.4 (1.0)	1.1 (−1.5 to 3.8)	42.7 (1.4)	1.3 (−2.4 to 5.2)	45.7 (1.3)	0.6 (−3.0 to 4.2)
Log_10_-transformed coefficient^c^		0.7 (−2.1 to 3.5)		0.8 (−2.8 to 4.4)		0.5 (−3.3 to 4.3)
BSI scores						
First quartile (0–1.16 μg/g)	48.8 (1.0)	Reference	48.9 (1.3)	Reference	48.7 (1.3)	Reference
Second quartile (1.17–1.80 μg/g)	51.7 (1.1)	2.9 (0.3 to 5.5)	53.9 (1.3)	5.0 (1.5 to 8.5)	49.2 (1.5)	0.5 (−3.2 to 4.3)*
Third quartile (1.81–2.96 μg/g)	50.9 (1.0)	2.2 (−0.4 to 4.7)	52.5 (1.3)	3.7 (0.1 to 7.2)	49.3 (1.4)	0.6 (−3.1 to 4.2)
Fourth quartile (> 2.96 μg/g)	50.8 (1.0)	2.1 (−0.5 to 4.6)	50.4 (1.4)	1.5 (−2.2 to 5.2)	51.0 (1.3)	2.3 (−1.2 to 5.8)
Log_10_-transformed coefficient^c^		1.4 (−1.3 to 4.1)		0.9 (−2.7 to 4.6)		1.8 (−1.7 to 5.2)

a Adjusted for maternal age (< 25, 25–34, and ≥ 35 years), child sex, race (white and nonwhite), maternal education (< 12 years, 12 years, > 12 years), household income (< $40,000, $40,000 to < $80,000, ≥ $80,000), HOME score (< 35, 35 to < 40. and ≥ 40), marital status (married vs. unmarried), and maternal depression during pregnancy (minimal/mild vs. moderate/ severe).

b Adjusted for maternal age (< 25, 25–34, and ≥ 35 years), race (white and nonwhite), maternal education (< 12 years, 12 years, > 12 years), household income (< $40,000, $40,000 to < $80,000, ≥ $80,000), HOME score (< 35, 35 to < 40, and ≥ 40), marital status (married vs. unmarried), and maternal depression during pregnancy (minimal/mild vs. moderate/severe).

c Adjusted for log_10_-transformed creatinine concentration, maternal age (< 25, 25–34, and ≥ 35 years), race (white and nonwhite), maternal education (< 12 years, 12 years, > 12 years), household income (< $40,000, $40,000 to < $80,000, ≥ $80,000), HOME score (< 35, 35 to < 40, and ≥ 40), marital status (married vs. unmarried), and maternal depression during pregnancy (minimal/mild vs. moderate/severe).

* *p*-Value < 0.10 for the interaction term between BPA concentration and child sex.

** *p*-Value < 0.05 for the interaction term between BPA concentration and child sex.
